# Measuring the Cognitive Workload During Dual-Task Walking in Young Adults: A Combination of Neurophysiological and Subjective Measures

**DOI:** 10.3389/fnhum.2020.592532

**Published:** 2020-11-20

**Authors:** Isabelle Hoang, Maud Ranchet, Romain Derollepot, Fabien Moreau, Laurence Paire-Ficout

**Affiliations:** Transport, Health, Safety Department, Laboratory Ergonomics and Cognitive Sciences Applied to Transport, Univ Gustave Eiffel, Univ Lyon, Lyon, France

**Keywords:** cognitive workload, dual-task walking, young adults, fNIRS, NASA-TLX questionnaire

## Abstract

**Background:** Walking while performing a secondary task (dual-task (DT) walking) increases cognitive workload in young adults. To date, few studies have used neurophysiological measures in combination to subjective measures to assess cognitive workload during a walking task. This combined approach can provide more insights into the amount of cognitive resources in relation with the perceived mental effort involving in a walking task.

**Research Question:** The objective was to examine cognitive workload in young adults during walking conditions varying in complexity.

**Methods:** Twenty-five young adults (mean = 24.4 ± 5.4) performed four conditions: (1) usual walking, (2) simple DT walking, (3) complex DT walking and (4) standing while subtracting. During the walking task, mean speed, cadence, stride time, stride length, and their respective coefficient of variation (CV) were recorded. Cognitive workload will be measured through changes in oxy- and deoxy-hemoglobin (ΔHbO_2_ and ΔHbR) during walking in the dorsolateral prefrontal cortex (DLPFC) and perceived mental demand score from NASA-TLX questionnaire.

**Results:** In young adults, ΔHbO_2_ in the DLPFC increased from usual walking to both DT walking conditions and standing while subtracting condition. ΔHbO_2_ did not differ between the simple and complex DT and between the complex DT and standing while subtracting condition. Perceived mental demand gradually increased with walking task complexity. As expected, all mean values of gait parameters were altered according to task complexity. CV of speed, cadence and stride time were significantly higher during DT walking conditions than during usual walking whereas CV of stride length was only higher during complex DT walking than during usual walking.

**Significance:** Young adults had greater cognitive workload in the two DT walking conditions compared to usual walking. However, only the mental demand score from NASA-TLX questionnaire discriminated simple from complex DT walking. Subjective measure provides complementary information to objective one on changes in cognitive workload during challenging walking tasks in young adults. These results may be useful to improve our understanding of cognitive workload during walking.

## Background

Walking is a complex human activity that requires coordinated and controlled movement, dynamic stability, motor and cognitive functions for safe ambulation. Walking while performing a secondary task [i.e., dual-task (DT) walking], as speaking, searching for an itinerary, or avoiding obstacles creates a complex situation which can vary the cognitive workload. Based on the idea that the individual's attentional capacity is limited (Paas and Van Merriënboer, 1994) have defined two components of the cognitive workload: the mental load and the mental effort. The mental load refers to the intrinsic characteristics of the task such as complexity of a task and/or information presentation and instruction format. The mental effort refers to the amount of cognitive resources allocated to perform the task. Several studies have shown that an increase of cognitive workload during walking led to a decrease of gait performance (Al-Yahya et al., 2011). Young adults showed decreased gait speed during walking while counting forward, subtracting or texting on a mobile phone (Mirelman et al., 2014; Schabrun et al., 2014; Plummer et al., 2015). Decreased gait speed was also observed when priority was given to the cognitive task, such as a verbal fluency task (Yogev-Seligmann et al., 2012). Yet, in context of DT walking, gait variability in young adults seems to be task dependent. It appeared that walking while texting was the most challenging DT, leading to greater gait variability in young adults. However, stride time variability did not increase from usual walking to walking while counting backward or walking while subtracting (Mirelman et al., 2014). It is therefore difficult to know exactly what is the impact of a dual-task on the walking performance in young adults. A more comprehensive assessment of cognitive workload may help to better understand this issue. Moreover, being able to properly identify the level of cognitive workload during walking in young adults would help to provide clinical and future research recommendations.

Several neurophysiological measures can be used to assess cognitive workload during a walking task. An approach to deduce neuronal activity during walking is to ask participants to imagine themselves performing a walking task, using functional magnetic resonance imagery (fMRI) (Hamacher et al., 2015). Although fMRI has adequate spatial resolution, it remains unclear how to ascertain whether subjects actually perform imagery of gait or not. Measuring brain activity in natural environments may be enabled by the electroencephalography (EEG) and functional near-infrared spectroscopy (fNIRS), which are non-invasive, portable, and relatively cost-effective methods. EEG records the integrated and synchronized activity of pyramidal neurons in the cortex (Berger et al., 2019). EEG biomarkers such as event-related potential (ERP) (i.e., N200 or P300) or spectral power analyses may serve as good indicators of cognitive workload while individuals perform the walking task (Shaw et al., 2018). Young adults showed an increase in cognitive workload, as indexed by an increase in gamma power over the frontal cortex when walking while performing a serial subtraction and finger tapping tasks (Marcar et al., 2014) or go/ no go task (De Sanctis et al., 2014). Another study found that young adults had lower alpha activity in frontal brain areas during DT walking (Beurskens et al., 2016). Although EEG has the advantage to have a good temporal resolution, the spatial resolution is relatively low. In addition, the presence of noise due to motion artifacts may affect data quality. To provide information regarding the spatial location of the recorded activity, fNIRS is more appropriate than EEG, with a very high experimental flexilibility (Berger et al., 2019; Quaresima and Ferrari, 2019). fNIRS measures changes in regional cerebral blood flow, cortical oxygenated hemoglobin (ΔHbO_2_) and deoxygenated hemoglobin (ΔHbR) induced by neuronal activation. Previous research showed that younger adults had an increase of oxygenated hemoglobin level in the prefrontal cortex during walking under DT conditions as compared to normal walking (Holtzer et al., 2011; Mirelman et al., 2014, 2017; Lu et al., 2015). For instance, different cognitive tasks were used such as n-back (Lin and Lin, 2016), talking (Holtzer et al., 2011), counting (Mirelman et al., 2014, 2017), or subtraction tasks (Hill et al., 2013; Meester et al., 2014; Mirelman et al., 2014, 2017; Lu et al., 2015). These cognitive tasks have in common the fact that they involve executive functions, which have been shown to be strongly linked to prefrontal cortex, particularly the dorsolateral prefrontal cortex (Kane and Engle, 2002). In this study, the cerebral activity of the dorsolateral prefrontal cortex while walking will be assessed to inform about the mental effort, meaning the amount of cognitive resources allocated to perform the task.

Another way to assess the level of cognitive workload is to ask people directly how they feel after performing walking through a questionnaire. A subjective measure may add complementary information to an objective one. Compared to neurophysiological tools, a questionnaire is cost-effective and easy to administer which makes it accessible for a large number of clinicians. An appropriate measure of the cognitive workload using an easy and quick questionnaire may lead to an expansion of its use. Knaepen et al. found that the subjective score on the mental demand subscale of the NASA-RTLX questionnaire was very sensitive to an increase of cognitive workload during walking in young adults (Knaepen et al., 2015). To date, only a few studies have used subjective measures to assess cognitive workload during a walking task (Popova-Dlugosch et al., 2013; Knaepen et al., 2015; Lin and Lin, 2016; Lin and Huang, 2017; Shaw et al., 2018; Pigeon et al., 2019) and very few studies have used a combined approach using neurophysiological and subjective measures to assess cognitive workload during walking (Knaepen et al., 2015; Lin and Lin, 2016; Shaw et al., 2018). Shaw et al. (2018) found that EEG temporal and spectral power analyses, as well as the mental demand subscale of the NASA-TLX questionnaire were good indicators of changes in cognitive workload associated with the difficulty of a cognitive task independently of the executed conditions (i.e., seated or walking). Lin and Lin (2016) found a different result with the fNIRS: cerebral activity was significantly different between tasks, however it did not classify tasks according to the increasing difficulty. Thus, it remains unclear if the fNIRS measures are able to detect changes in cognitive workload between different difficulty levels of cognitive task.

The objective of this study was therefore to examine cognitive workload in young adults during walking conditions varying in complexity. Cognitive workload will be measured through changes in cerebral activity of the DLPFC, using the fNIRS and mental demand score from NASA-TLX questionnaire. Usual walking, simple and complex DT walking conditions are used to examine changes in cognitive workload.

The interest of using both physiological and subjective measures is to provide insights into the amount of cognitive resources allocated to perform the walking task in relation to the level of task difficulty felt by individuals. We hypothesize that cognitive workload in young adults will increase with the difficulty of the walking task. This increase in cognitive load should be observed with both physiological and subjective measures. Moreover, associations between subjective and neurophysiological measures will also be explored.

## Materials and Methods

### Participants

Twenty-five healthy young adults (mean = 24.36 ± 5.38; 9 females, years of education: 14.6 ± 2.1) were included in this study. Participants were recruited through advertisements in University of Lyon 2 and the French Institute of Science and Technology for Transport, Development and Networks. The study took place between June 18, 2018 and November 29, 2018. Young adults were included if they had no underlying neurological diseases or gait abnormalities that might interfere with walking. This study was approved by the local French Ethical Committee in March 9, 2018. All participants provided informed written consent.

### Protocol

**Figure 1:**
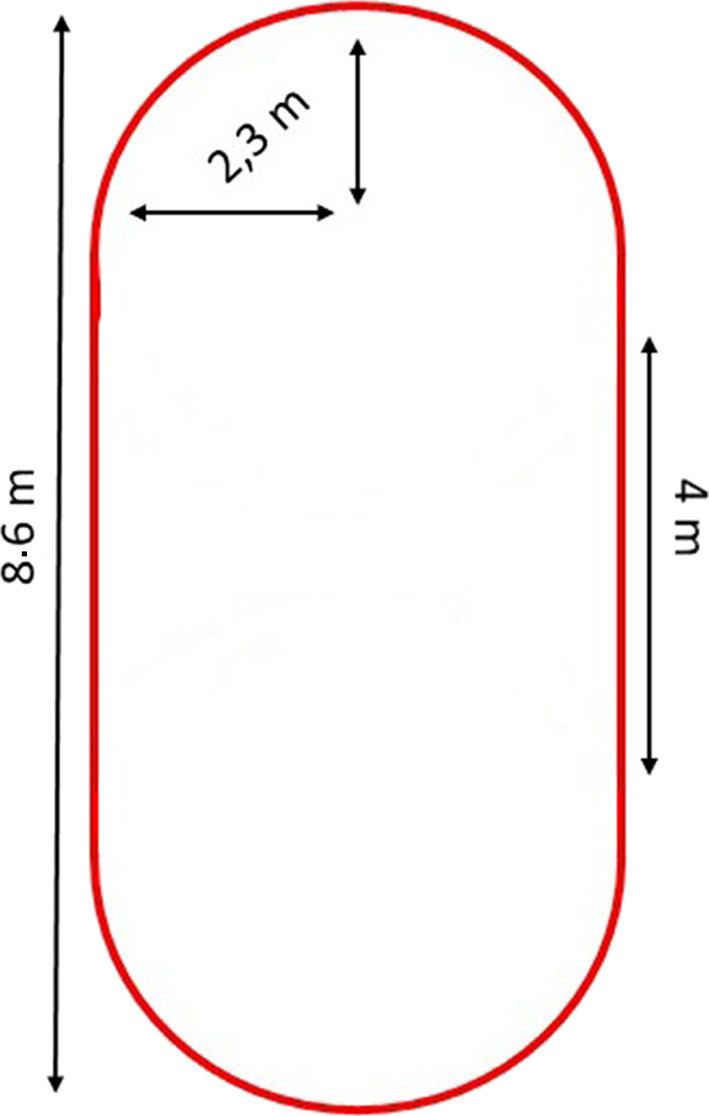
Walking path configuration.

Participants were equipped with the fNIRS system and two sensors on their shoes. They performed four conditions: (1) usual walking: walking in a self-selected comfortable speed; (2) simple DT: walking while counting forward from a three-digit number which was included to add a level of complexity; (3) complex DT: walking while subtracting 7 from a three-digit number, and (4) standing while subtracting 7 to determine whether HbO_2_ changes during walking while subtracting are mainly due to the demands of the subtraction task. The order of each condition was randomized between participants. For the DT conditions, no instructions of prioritization were given. During the standing while subtracting condition as well as the two DT walking conditions, the number of correct responses (cognitive performance) was also measured. The walking path dimensions are depicted in Figure 1. Each condition included 5 trials of 30 s. The duration of the rest period between trials varied from 25 to 35 s to diminish possible resonance effects (Herold et al., 2018). All conditions began with 45 s of quiet standing. The total duration of each condition was 6 min. Participants were allowed to take breaks between conditions.

### Gait Assessment

Gait parameters were recorded with two inertial foot-sensors (Physilog®5, Gait Up, Switzerland) including 3D accelerometer (up to 16 g), 3D gyrospcope (up to 2,000°/s), a memory, a battery and a microcontroller. The two sensors were connected by Bluetooth and were fixed on the shoes with a rubber clip. Mean speed (meter per second), stride length (meter) and cadence (number of step/minute), as well as their respective coefficient of variation (CV) were measured.

### Functional Near Infrared Spectroscopy

**Figure 2:**
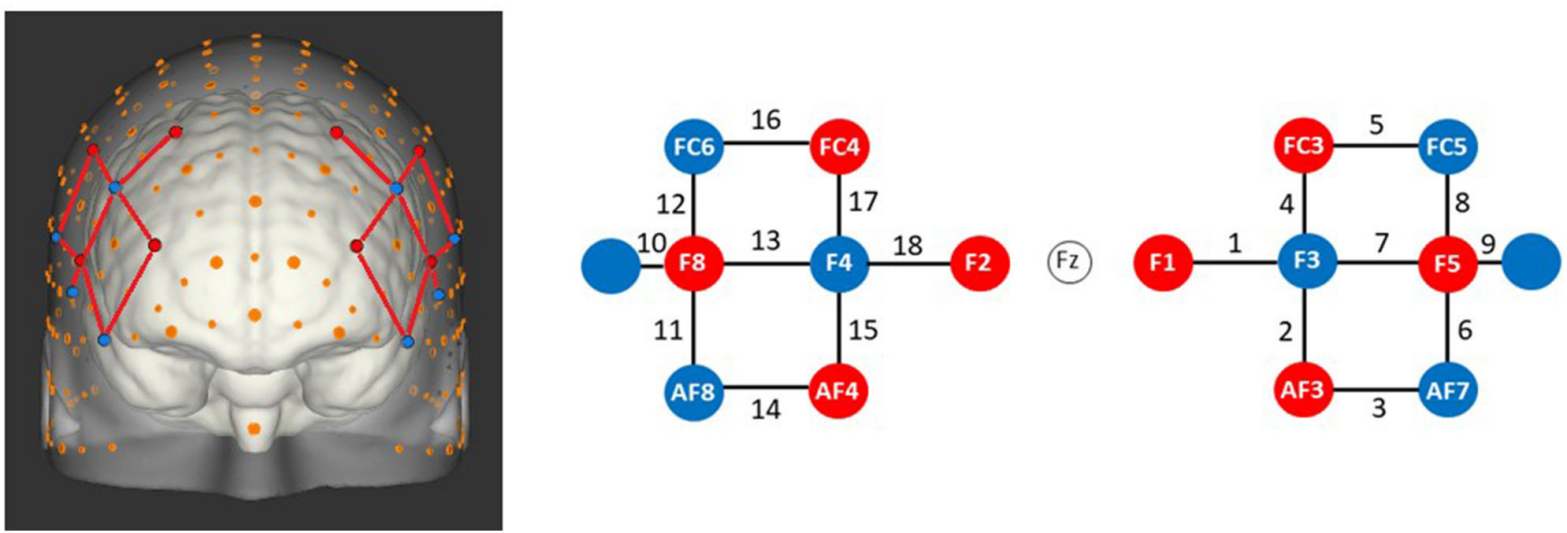
fNIRS montage measuring the prefrontal dorsolateral cortex according to the EEG 10-10 system. Red circles represent sources and blue circles represent detectors. Numbers represent channels.

Changes in HbO_2_ and HbR concentration (μmol/L) in the DLPFC were measured using a wireless continuous waves fNIRS device (NIRSport, NIRx Medical Technologies) with 16 channels. An increase of ΔHbO_2_ associated with a slight decrease of ΔHbR reflects a functional activation for the task (Villringer and Chance, 1997). Optodes (8 sources and 8 detectors) were separated by ~30 mm and were placed on the DLPFC according to the modified international EEG 10-10 system (Chatrian et al., 1985) (see Figure 2). Two short separation channels with an interoptode distance of 15 mm were used in order to remove hemodynamic changes in superficial tissue layers. Sources and detectors were fixed on fNIRS caps which are themselves adapted to the size of the participant's head (i.e., circumference of 54, 56, 58 cm). The near infrared light was emitted by sources with wavelengths of 760 and 850 nm at a sampling rate of 7.81 Hz. An overcap was used to prevent ambient-light contamination. Raw intensities were recorded using the software provided by the manufacturer (NIRStar, version 15.1 and 15.2).

### Subjective Assessment

The National Aeronautics and Space Administration Task Load Index (NASA-TLX) questionnaire was used to assess participants' subjective level of workload (Hart and Staveland, 1988; Cegarra and Morgado, 2009). After each condition, participants were asked to rate five of its six subscales (mental demand, physical demand, effort, performance, and frustration) on a scale ranging from 0 (very low) to 100 (very high) (10-point scale). Temporal demand subscale was not considered because it is not appropriate in the context of the present walking experiment. For the purpose of this study, only the mental demand subscale, which best assesses the cognitive workload was analyzed (Hart, 2006; Young et al., 2008). Participants were asked to assess the mental demand required to perform each condition. The weighting process was not applied (the Raw-TLX version) but remains as sensitive as the NASA-TLX questionnaire (Byers et al., 1989).

### Data Processing

The data recorded from fNIRS were analyzed using the open-source software Homer 2. Homer 2 is a set of Matlab scripts used to analyze fNIRS data (version 2.8) (R2018b, MathWorks). A processing chain was applied to each dataset to recover data free of artifacts or noise. The first processing step was to convert raw data into optical density. Then, a low pass filter with a cut-off frequency of 0.1 Hz was applied to attenuate respiration and cardiac activity and high frequency noise. The next step was a motion artifact correction using wavelet-based filters (iqr = 1.5) (Molavi and Dumont, 2012; Brigadoi et al., 2014) and principal component analysis (nSV = 0.8) (Wilcox et al., 2005; Cooper et al., 2012). After motion correction, optical density was converted into relative concentration changes using the modified Beer-Lambert law with an age-dependent differential path length factors (DPF) value of six (Herold et al., 2018). Finally, contribution of short separation channels was removed from the signal using a Kalman filter dynamic estimator (Gagnon et al., 2012). Mean relative changes in HbO_2_ and HbR concentrations were obtained for each channel using the last 5 s of the resting state before each condition as a baseline and 30 s after the beginning of the task, using the block-average method. All trials were averaged per condition.

### Statistical Analysis

Kolmogorov-Smirnov tests were used to determine the normality of variables. Repeated measures analyses of variance (ANOVA) with condition (usual walking, simple DT walking, complex DT walking) as within-subject factor were performed on gait parameters. Repeated measures ANOVA with condition (usual walking, simple DT walking, complex DT walking, standing while subtracting) were also performed on fNIRS data (ΔHbO_2_ and ΔHbR) and mental demand score. A mental demand score is missing for one participant on one condition. Therefore, scores from this participant were removed from the repeated measures ANOVA on mental demand score. Repeated measures ANOVA with condition (simple DT walking, complex DT walking, standing while subtracting) was also performed on cognitive performance. For each ANOVA, effect sizes (η2) were reported and were interpreted as small (0.02), medium (0.13), and large (0.26) (Bakeman, 2005). Bonferroni correction for multiple comparisons was applied during the *post-hoc* analyses. Pearson or Spearman rank correlations were conducted to investigate associations between measures of cognitive workload (ΔHbO_2_ and mental demand score) and behavioral performance (gait and cognitive performance) within condition. Spearman or Pearson correlations (ρ or r, respectively) were considered weak below 0.10, moderate between 0.10 and 0.49 and strong between 0.50 and 1.00 (Cohen, 1992). *P* < 0.05 were considered significant. All statistical analyses were conducted using SPSS software, version 26.

## Results

### Gait Performance

There was a main effect of condition on stride time [*F*_(2, 48)_ = 22.685, *p* < 0.001, η^2^ = 0.486], gait speed [*F*_(2, 48)_ = 35.583, *p* < 0.001, η^2^ = 0.597], cadence [*F*_(2, 48)_ = 28.492, *p* < 0.001, η^2^ = 0.543], and stride length [*F*_(2, 48)_ = 29.014, *p* < 0.001, η^2^ = 0.547]. *Post-hoc* tests showed that stride time was longer during DT walking conditions (simple and complex). Gait speed and cadence were slower and stride length was shorter during DT walking conditions than during usual walking (see Table 1). Stride time was longer in complex DT walking compared to simple DT walking. Gait speed, cadence and stride length were significantly altered in complex DT walking compared to simple DT walking.

**Table 1:** Gait parameters for each walking task in young adults (mean, SD).

	**Usual walking**	**Simple-DT**	**Complex-DT**	Pairwise comparison^*^		
				**Usual walking—Simple-DT**	**Usual walking—Complex-DT**	**Simple DT—Complex-DT**
Speed (m/s)	1.14 ± 0.13	1.01 ± 0.14	0.90 ± 0.20	*p* < 0.001	*p* < 0.001	*p* < 0.001
Cadence (step/min)	103.04 ± 8.01	95.45 ± 9.57	88.28 ± 13.85	*p* < 0.001	*p* < 0.001	*p* < 0.001
Stride length (m)	1.30 ± 0.12	1.25 ± 0.12	1.19 ± 0.14	*p* < 0.001	*p* < 0.001	*p* < 0.001
CV of speed (%)	7.42 ± 1.99	9.31 ± 3.01	9.78 ± 3.15	*p* < 0.05	*p* < 0.01	NS
CV of cadence (%)	3.53 ± 1.15	5.12 ± 2.45	5.52 ± 2.71	*p* < 0.05	*p* < 0.01	NS
CV of stride length (%)	5.45 ± 1.41	6.18 ± 1.03	6.92 ± 1.60	NS	*p* < 0.01	NS

*Values are expressed as mean ± SD*.

* *P-value adjusted for Bonferroni correction*.

*NS, non-significant*.

There was also a main effect of condition on CV of stride time [*F*_(2, 48)_ = 9.608, *p* < 0.001, η^2^ = 0.486], CV of gait speed [*F*_(2, 48)_ = 8.255, *p* < 0.01, η^2^ = 0.256], CV of cadence [*F*_(2, 48)_ = 10.463, *p* < 0.001, η^2^ = 0.286], and CV of stride length [*F*_(2, 48)_ = 10.072, *p* < 0.001, η^2^ = 0.296]. CV of stride time, CV of gait speed and CV of cadence were higher during DT walking conditions (simple and complex) than during usual walking. CV of stride length was only significantly higher during complex DT walking than during usual walking. For all CV of gait parameters, no significant differences between complex DT and simple DT walking conditions were found.

### Cognitive Performance

Main effect of condition was found on cognitive performance [*F*(2, 48) = 173. 32, *p* < 0.001, η^2^ = 0.88]. Pairwise comparisons showed no significant differences in the number of correct responses during subtraction task between standing and walking conditions (see Table 2). However, the number of correct responses were significant higher during simple DT walking than the two other conditions.

**Table 2:** Number of correct responses for each task in young adults (mean, SD).

				Pairwise comparison^*^		
	**Standing**			**Standing while**	**Standing while**	**Complex DT - Simple DT**
	**while**			**subtracting—**	**subtracting—**	
	**subtracting**	**Complex DT**	**Simple DT**	**Complex DT**	**Simple DT**	
Number of correct responses	7.11 ± 3.98	6.56 ± 4.20	25 ± 7.71	NS	*p* < 0.001	*p* < 0.001

*Values are expressed as mean ± SD*.

* *P-value adjusted for Bonferroni correction*.

*NS, non-significant*.

### Cerebral Activity

**Figure 3:**
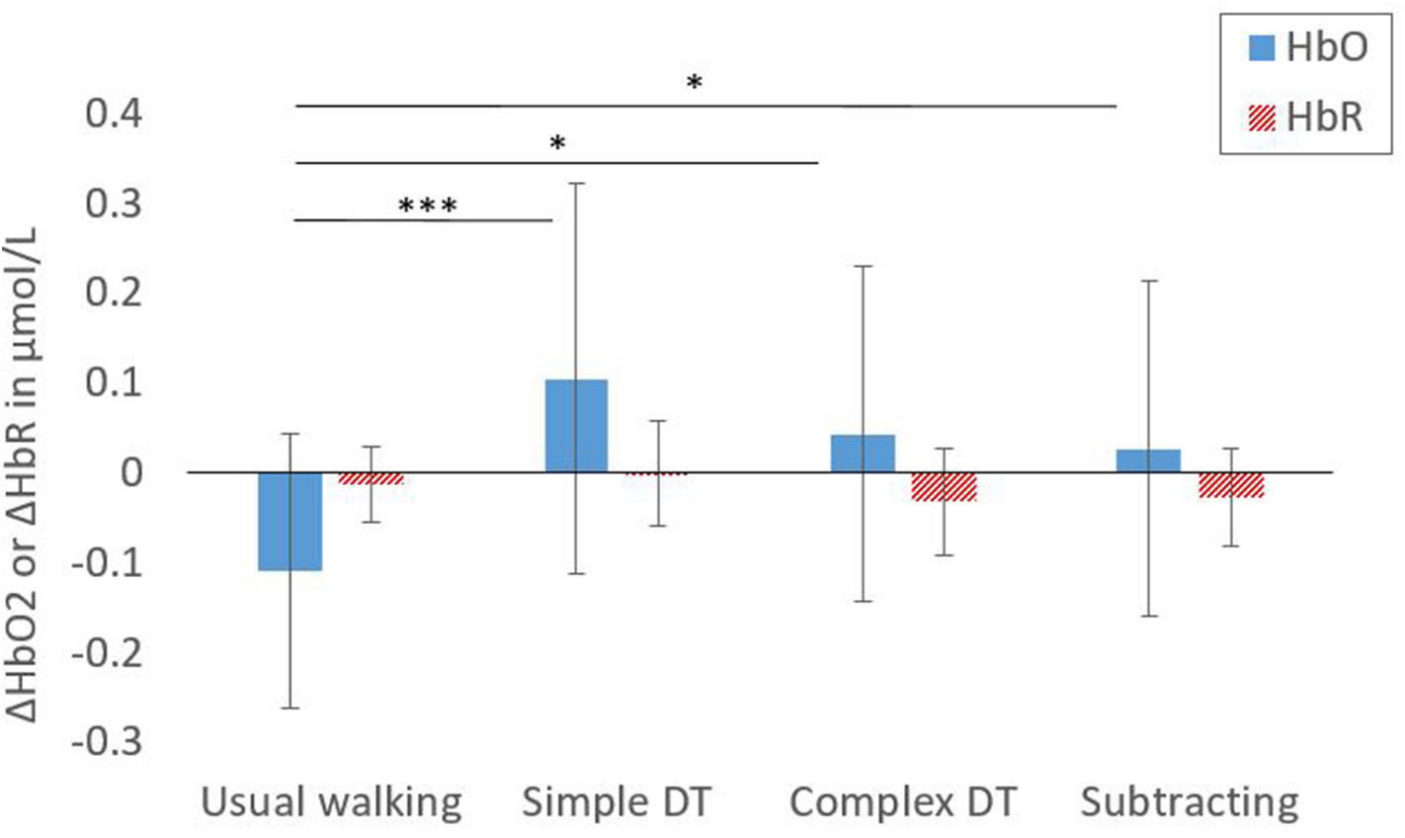
Mean values for ΔHbO2 and ΔHbR in the dorsolateral prefrontal cortex. Significance level between conditions: *p < 0.05; ***p < 0.001. Error bars represent ±1 SD.

Since no significant differences in ΔHbO_2_ and ΔHbR were observed between left and right hemispheres of the DLPFC (*p* = 0.133, *p* = 0.709, respectively), ΔHbO_2_ and ΔHbR for each channel were then averaged for the whole DLPFC. A main effect of condition on ΔHbO_2_ was found [*F*_(3, 72)_ = 12.51, *p* < 0.001, η^2^ = 0.289] (Figure 3). Compared to usual walking, ΔHbO_2_ were significantly higher during simple and complex DT walking conditions and during standing while subtracting. No main effect of condition on ΔHbR was found [*F*_(3, 72)_ = 2.701, *p* > 0.05, η^2^ = 0.101].

### Subjective Measure of Mental Demand

**Figure 4:**
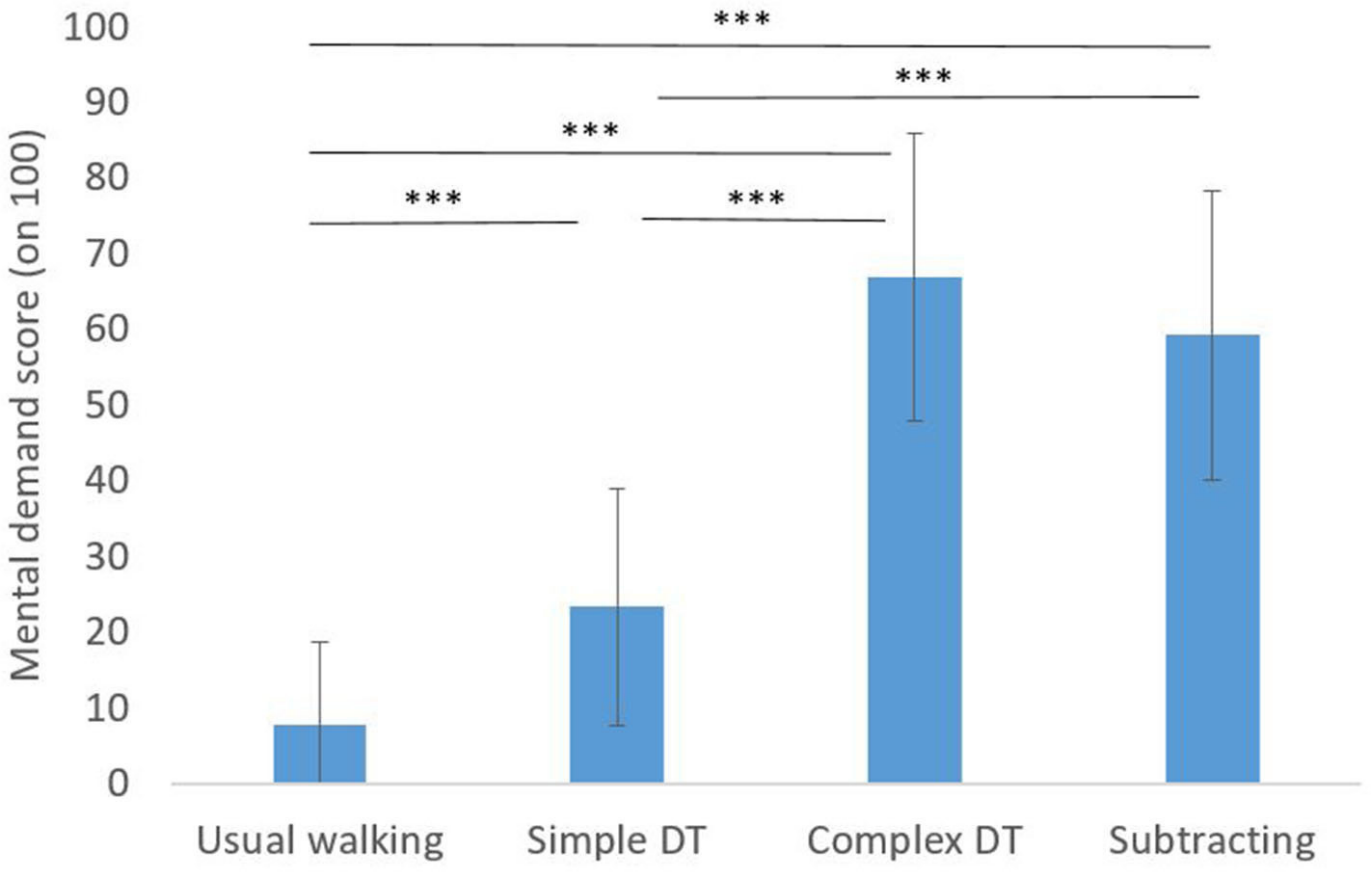
Mean values of the mental demand subitem of the NASA-TLX questionnaire. Significance level between conditions: ***p < 0.001 Error bars represent ±1 SD.

A main effect of condition on mental demand score was found [*F*_(3, 69)_ = 122.412, *p* < 0.001, η^2^ = 0.842]. Mental demand scores were significantly greater after simple and complex DT walking conditions, and after standing while subtracting as compared to usual walking (Figure 4). Also, the mental demand scores felt after the standing while subtracting condition was significantly greater than after usual walking and simple DT walking. However, no significant differences were found in the mental demand score between complex DT walking and standing while subtracting (*p* > 0.05).

### Associations Between Cognitive Workload Measures

No significant correlations between cerebral activity in the DLPFC and the mental demand score were found during usual walking, simple DT, and complex DT walking conditions (*p* > 0.05 for all conditions).

### Associations Between Cognitive Workload Measures and Behavioral Performance

No significant correlations between cognitive workload measures (cerebral activity and mental demand score) and behavioral performance (gait and cognitive performance) during usual walking, simple DT, and complex DT walking conditions were found (*p* > 0.05 for all conditions).

## Discussion

Main findings showed that young adults had greater cognitive workload, assessed by cerebral activity in the DLPFC and mental demand score of the NASA-TLX questionnaire, during both simple and complex DT walking conditions than during usual walking. Interestingly, changes in cerebral activity in the DLPFC did not differ between the two DT conditions whereas young adults declared feeling a greater mental demand after the complex DT walking than after simple DT walking. Moreover, walking performance of young adults decreased as the task becomes complex. To our knowledge, only one study in young adults used a combined approach, using fNIRS and the NASA-TLX questionnaire to assess cognitive workload during walking (Lin and Lin, 2016). Our findings could improve detection of cognitive workload changes during walking under different levels of difficulty in young adults.

In this study, the increase in DLPFC activity during both simple and complex DT walking conditions compared to usual walking, is in accordance with previous findings in young adults (Holtzer et al., 2011; Doi et al., 2013; Mirelman et al., 2014; Beurskens et al., 2016; Fraser et al., 2016; Lin and Lin, 2016; Metzger et al., 2017). This suggests that individuals needed to recruit a greater amount of cognitive resources to perform simultaneously the walking and the cognitive tasks (Mirelman et al., 2014; Lin and Lin, 2016; Shaw et al., 2018). However, cerebral activity, performance and mental demand did not differ in the subtraction task while either walking or standing. Consequently, the increase of cognitive workload during complex DT walking as compared to usual walking is mainly due to the addition of the subtraction task.

Also, cerebral activity in the DLPFC did not differ between the two levels of difficulty of DT walking. This could be due to the fact that young participants did not recruit additional cognitive resources to perform the complex DT walking compared to the easy DT walking. It is also possible that the complex task (walking while subtracting) was too difficult, resulting in disengagement of some participants. In this condition, the cognitive workload was not measurable as few cognitive resources were allocated to perform the task. Therefore, the activation level remained unchanged between the two simple and complex conditions. However, the fact that subtraction performance did not differ between walking while subtracting and standing while subtracting suggests that participants were engaged in the complex task, at least as engaged as in the standing condition. Furthermore, in this study, we only considered the mean HbO_2_ as an indicator of cognitive workload. Other indicators (i.e., maximal HbO_2_, slope, time to peak,…) might be used to assess cognitive workload during DT walking as it is done in different studies (Menant et al., 2020).

The lack of changes in cognitive workload may also suggest that the measure of cerebral activity in the DLPFC is not sufficient to differentiate between different workloads levels during a dual-task walking situation, involving arithmetic tasks. This is not consistent with a previous study using EEG biomarkers which showed changes in cognitive workload during DT walking conditions varying in difficulty (Shaw et al., 2018). Divergences between studies may be due to the nature of cognitive task [counting forward in the present study vs. visual task (Shaw et al., 2018)] during walking. Moreover, it is possible that walking while counting forward may have affected our findings on cerebral activity. It involved more rapid and continuous talking than the subtracting task that can create artifacts in the measure. This may therefore influence HbO_2_ levels within the cortex and partly represent speech demands inherent to the walking while counting forward condition (Scholkmann et al., 2013; Schecklmann et al., 2017).

Finally, findings obtained with fNIRS and the mental demand score as well as the lack of significant correlation between subjective mental demand score and neurophysiological measures showed that the two measures used in this study provide complementary information regarding the cognitive workload. An interpretation of these findings could be that the fNIRS measures the mental effort i.e., the amount of cognitive resources allocated to perform the task whereas the NASA-TLX questionnaire assesses both the mental load and the mental effort, referred to by Paas and Van Merriënboer (1994). When participants are asked to rate their mental effort with NASA-TLX questionnaire, it is not clear whether they were really able to discriminate between the two components of mental load (difficulty of the task vs. amount of resources).

As the complexity of the walking task increased, speed, cadence, and stride length decreased in young adults, which is consistent with previous studies (Mirelman et al., 2014; Beurskens et al., 2016). Conversely, Hill et al. (2013) did not find the same results: no differences in mean gait speed were observed between simple and complex DT walking. Divergences between the two studies may be explained by the differences in methodologies. In Hill et al. study, half of the participants completed the simple task and the other half the complex one, whereas all participants completed both tasks in the present study.

Young adults also showed a higher gait variability during both simple and complex DT walking conditions than during usual walking, suggesting that the DT, regardless of its nature, influence walking stability. Results were different from those of Mirelman's study (Mirelman et al., 2014) using a similar methodology in which gait variability, indexed by CV of stride time did not differ between the walking conditions. It is possible that our path configuration was more complex than the straight line used in their study. During each walking condition, we observe that our participants had a greater stride time variability than in Mirelman's study (e.g., during usual walking: 2.35 ± 0.50; Mirelman et al., 2014 vs. 3.68 ± 1.29).

Young adults maintained good subtraction performance and have poorer gait performance during complex DT walking. Although no associations were found between cognitive workload measures and behavioral performance, this result suggests that young adults may prioritize the subtraction task at the expense of the walking task, as shown in previous studies (Yogev-Seligmann et al., 2012; Plummer et al., 2015; Raffegeau et al., 2018). Yogev-Seligmann et al. (2012) suggested that young adults may have sufficient postural reserve to focus on the cognitive task.

This study has some limitations. The small sample size limits the generalizability of the results. One methodological limitation is the lack of one control condition i.e., standing while counting condition, to determine whether the increase of ΔHbO_2_ in simple DT walking is due to muscle movements provoked by counting aloud (Schecklmann et al., 2017). Another limitation of the present study is that only the activity of DLPFC cortex is recorded. Further studies should assess other cerebral regions (e.g., occipital, premotor and parietal regions) while walking to provide a greater understanding of the contribution of brain areas to walking and dual tasking (Stuart et al., 2019).

This study showed that measures of cerebral activity and mental demand score provide complementary information about the cognitive workload during a walking task in young adults. Cerebral activity did not differ between the simple (counting forward) and complex (subtracting) DT. By contrast, young adults declared feeling greater mental effort after the complex DT walking as compared to the simple DT walking. This research suggests that subjective measures should be used in combination to objective measures to improve the understanding of cognitive workload during walking under different levels of complexity in young adults. It may allow researchers and clinicians to better address the possible difficulties of gait impairments. Future studies investigating walking in the population of older adults are warranted.

## Data Availability Statement

The raw data supporting the conclusions of this article will be made available by the authors, without undue reservation.

## Ethics Statement

The studies involving human participants were reviewed and approved by Comité de Protection des Personnes Nord-Ouest III. The patients/participants provided their written informed consent to participate in this study.

## Author Contributions

IH, MR, and LP-F conceived the study design and contributed to data interpretation and the manuscript writing. IH performed, conducted the experiment, statistical analyses, and drafted the manuscript. IH, RD, and FM processed the data. All authors contributed to the article and approved the submitted version.

## Conflict of Interest

The authors declare that the research was conducted in the absence of any commercial or financial relationships that could be construed as a potential conflict of interest.
